# The cranial nerves: extensions of the central nervous system or components of the peripheral nervous system - how should we evaluate them?

**DOI:** 10.1590/0100-3984.2021.54.3e1

**Published:** 2021

**Authors:** Fernanda Rueda-Lopes

**Affiliations:** 1 Adjunct Professor at the Universidade Federal Fluminense (UFF), Niterói, RJ, Brazil, and Radiologist for the Group DASA, Rio de Janeiro, RJ, Brazil. Email: frueda81@hotmail.com.

The 12 cranial nerves are generally considered to be components of the peripheral nervous system. However, the first and second cranial nerves (olfactory and optic nerves, respectively) are considered to be extensions of the central nervous system, because they are myelinated by oligodendrocytes, whereas the 10 other cranial nerves are myelinated by Schwann cells^(1)^. The other cranial nerves originate from gray matter nuclei located in the brainstem, with the exception of the eleventh cranial nerve (accessory nerve), whose nucleus is in the spinal cord^(2)^. From the brainstem, they have a cisternal trajectory (i.e., they cross the basal cisterns, immersed in cerebrospinal fluid) within the arachnoid membrane, at which point they are considered roots; when they pass through that barrier, the perineurium develops and they become nerves^(1)^.

After exiting through the skull base foramina, the cranial nerves carry motor stimuli and return sensory afferents, as well as autonomic information, from structures of the head, face, and neck^(2)^. Therefore, these fine structures, which have a complex path and are difficult to identify on imaging examinations, play key roles in the development of several diseases. Therefore, magnetic resonance imaging and computed tomography are crucial to the anatomical and pathological evaluation of these nerves.

Magnetic resonance imaging of the brain is considered the gold standard for evaluating diseases of central nervous system^(3-5)^, as well as those of the cranial nerves. The use of three-dimensional (3D) steady-state free precession (SSFP) sequences, which are heavily T2-weighted, provides great contrast between the fine structures of the cranial nerves and the surrounding cerebrospinal fluid, allowing their cisternal segments to be identified^(2)^. However, additional sequences are needed in order to assess the pattern of nerve disease, including contrast-enhanced T1-weighted sequences for the assessment of neuritis, as has been observed in the current pandemic, caused by infection with severe acute respiratory syndrome coronavirus 2^(6)^. Such sequences can also be used for the assessment of infectious processes and tumors, as well as for the assessment of congenital, traumatic, and vascular diseases of the cranial nerves, as discussed and illustrated in the pictorial essay authored by Dalaqua et al.^(7)^, published in this issue of Radiologia Brasileira. The muscle fiber integrity in the muscle groups innervated by a given nerve can be evaluated in T1-weighted sequences, and short-tau inversion-recovery sequences can be used in order to map the extent of denervation, thus allowing the assessment not only of the anatomy of a nerve but also of its function^(2,8)^.

Computed tomography of the skull base is also useful for assessing the course of the cranial nerves. The skull base foramina are the exits through which the nerves pass on their way to the structures of the face and neck. Knowledge of this anatomy and comparison with the contralateral side allow the assessment of bone remodeling and irregularities, which can be related to conditions such as nerve sheath tumors^(2)^. One thing that should be debated and further clarified is the radiological approach stipulated in the examination protocol. Due to bureaucratic and financial constraints, we seek to limit the examinations to a specific body segment, such as the skull, face, and orbits. Therefore, when there is a request for magnetic resonance imaging of the skull to evaluate paralysis of the third cranial nerve (oculomotor nerve), do we simply choose to include an SSFP sequence to evaluate the cisternal portion of this nerve, or do we decide to perform a complete analysis of its course, including sequences aimed at evaluating the cavernous sinus and orbit? We should reflect upon whether we really should work within the limits of the examination request or should expand our perspective and follow the entire path of the nerve in question.

Another way to assess the peripheral nervous system is with neurography^(8,9)^. Using several 3D sequences, including 3D fast imaging with steady-state precession and 3D short-tau inversion-recovery sequences, together with intravenous contrast administration, the neurography examination allows the evaluation of the course of the nerve and its thickness, as well as the organization of the fascicles, signal intensity, and pattern of contrast enhancement. The use of neurography has recently been described for investigation of the nerves of the face and neck, which originate from the intracranial nerves, allowing the identification of features such as the mandibular and maxillary portions of the fifth cranial (trigeminal) nerve^(8)^.

In conclusion, the study of the cranial nerves is a complex topic, as demonstrated in the pictorial essay crafted by Dalaqua et al.^(7)^, given that there are multiple causes of injury, as well as multiple means of evaluating those causes. Nevertheless, because of advances in magnetic resonance imaging with SSFP sequences and the advent of neurography, neuroimaging now makes a major contribution to this field of investigation and diagnosis.
